# Bleeding Lymph Node Mass in a Postoperative Case of Thyroid Carcinoma: Transarterial Embolization to the Rescue

**DOI:** 10.7759/cureus.86214

**Published:** 2025-06-17

**Authors:** Biswajit Sahoo, Arunprakash Pitchaimuthu, Phanindra K Swain, Manoj Nayak

**Affiliations:** 1 Radiology, All India Institute of Medical Sciences, Bhubaneswar, Bhubaneswar, IND; 2 Surgical Oncology, All India Institute of Medical Sciences, Bhubaneswar, Bhubaneswar, IND

**Keywords:** embolization, interventional radiology, minimally invasive, nodal mass bleeding, thyroid carcinoma

## Abstract

Recurrent metastatic cervical lymph nodes are common in patients with thyroid cancer, even after standard treatments such as total thyroidectomy and lymph node dissection. However, skin ulceration accompanied by bleeding from these metastatic lymph nodes is extremely rare and is usually managed through surgical ligation. In certain cases, surgical ligation is nearly impossible due to limited access to the bleeding vessels caused by vascular involvement. Our patient is a 42-year-old male diagnosed with thyroid carcinoma two years ago who has received all standard treatment options. Subsequently, he developed a recurrent nodal mass in the neck region that was bleeding profusely and was successfully managed through transarterial embolization. Transarterial embolization is a minimally invasive and safe technique that can be utilized in challenging scenarios where surgical access to the bleeding vessels is limited due to vascular involvement.

## Introduction

Life-threatening bleeding in head and neck cancers can result from locally advanced ulcerating primary malignancies, direct tumour invasion into adjacent blood vessels, or carotid blowout syndrome, which is usually managed through surgery. The external carotid artery and its branches are pre-operatively embolised in highly vascular head and neck tumours to mitigate intraoperative bleeding, for managing carotid blowout syndrome and palliative bleeding in head and neck tumours [1]. Head and neck tumours with bleeding can be effectively treated by targeting bleeding vessels through endovascular intervention, supported by Rzewnicki et al.'s study of seventy-six patients [1]. Transarterial embolisation (TAE) is a minimally invasive procedure in which the blood supply to tumours is reduced using embolizing agents delivered through a catheter inserted via an arterial puncture in the skin. Neck lymph nodes are the most frequent site of recurrence after initial thyroid cancer treatment, especially for the papillary type [2]. However, skin ulceration with significant bleeding caused by a metastatic nodal neck mass is exceedingly rare [3]. Herein, we successfully managed a bleeding node with known thyroid carcinoma through transarterial embolization in a 40-year-old male patient.

## Case presentation

A 40-year-old male patient underwent an ultrasound at our institute for a swelling on the left side of the neck that had persisted for four months. The neck ultrasound revealed two nodules in the left lobe of the thyroid, measuring 3.2 x 4.5 cm and 2.2 x 2.0 cm, along with several left cervical lymph nodes. Fine needle aspiration cytology (FNAC) was performed on the thyroid nodules, which displayed features of thyroid carcinoma (Bethesda category VI). He was clinically staged as T3aN0M0. He underwent a total thyroidectomy with central and left lateral neck dissection in March 2022. A final diagnosis of poorly differentiated thyroid carcinoma was made on histopathology.

On immunohistochemistry, the tumour cells were diffusely and firmly positive for thyroid transcription factor 1 (TTF1), B cell leukemia/ lymphoma 2 (BCL2), tumour protein 53 (TP53)-70% (+3), focally and weakly positive for thyroglobulin (TG) and Cytokeratin 7 (CK7), while negative for calcitonin, carcinoembryogenic antigen (CEA), cytokeratin 20 (CK20), and cyclin D1. Neck nodes were free of metastasis. A whole-body iodine (WBI) scan in June 2022 revealed a 2.44% uptake in the thyroid bed, and the patient underwent radioactive iodine therapy (RAI) with iodine-131 (50 mCi). A post-RAI therapy WBI scan in July 2022 revealed no uptake or additional lesions.

In February 2024, he presented with left-sided neck swelling. On computed tomography (CT), multiple discrete to conglomerate neck nodes were noted on the left side, with a few metastatic lung nodules. A biopsy of neck nodes and lung nodules was done, which were positive for malignancy. He was planned for radiotherapy followed by lenvatinib; however, he could not turn up for the treatment due to financial constraints.

**Figure 1:**
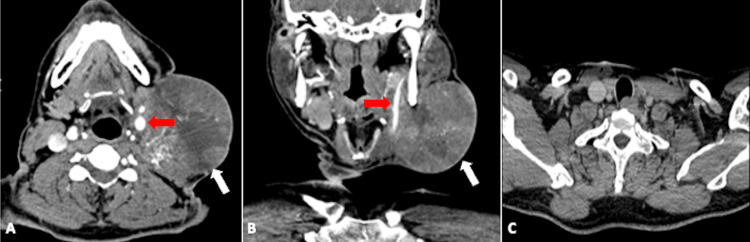
Contrast-enhanced CT scan of the patient with a bleeding nodal mass Contrast-enhanced CT scan axial (A) and coronal (B) images showing a nodal mass on the left side of the neck (white arrows) with encasement of the external carotid artery (red arrows). The nodal mass was closely abutting and displacing the internal carotid artery with an angle of contact of more than 270 degrees (not shown). Normal thyroid tissue is absent in the axial (C) image (postoperative).

In June 2024, he came to our emergency room with profuse bleeding from the neck mass. His haemoglobin was 7 g/dl (reference range 14-18 g/dl) at the presentation time. On examination, a solid, firm neck mass was palpated on the left side of the neck with ulceration of the overlying skin from which blood was oozing. A tight neck pressure dressing temporarily managed the bleeding, and subsequently, he received two units of blood transfusion. The patient was referred for contrast-enhanced CT, which showed multiple enlarged, discrete to conglomerated, highly vascular nodes on the left side of the neck with multiple feeders from the external carotid artery (ECA) (Figure 1).

**Figure 2:**
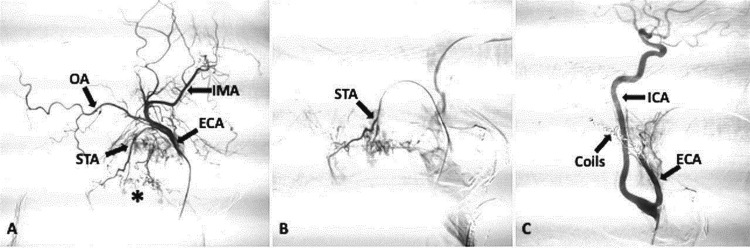
Digital subtraction angiography (DSA) images showed pre and post embolisation of bleeding nodal mass. (A) Pre-embolization: Sagittal digital subtraction angiography (DSA) image showing tumor blush (asterisk) in nodal mass. The nodal mass is primarily supplied by the occipital artery (OA) and superior thyroid artery (STA). A few small arterial twigs directly arising from the ECA are also supplying the nodal mass. The tip of the catheter is in the proximal ECA. (b) Pre-embolization: Sagittal DSA image showing superselective catheterization of the superior thyroid artery (STA) by using a microcatheter. (c) Post-embolization: Sagittal DSA image showing marked reduction in tumor blush after embolization with polyvinyl alcohol particles and coils. IMA: internal maxillary artery; ICA: internal carotid artery.

The nodal mass encased the proximal segment of the ECA and displaced the internal carotid artery (ICA) medially. The nodal mass was closely abutting and displacing the ICA with an angle of contact of more than 270 degrees. Vascular involvement created a nearly impossible situation for the surgeons to manage the bleeding, as access to the bleeding vessels was limited. The patient was shifted to TAE after an interventional radiology consultation. Selective catheterisation of the left ICA and ECA was done, followed by a contrast angiogram, which confirmed the presence of multiple feeders from the ECA and its branches. The nodes were predominantly supplied by ECA's superior thyroid artery and occipital branches (Figure 2).

**Figure 3:**
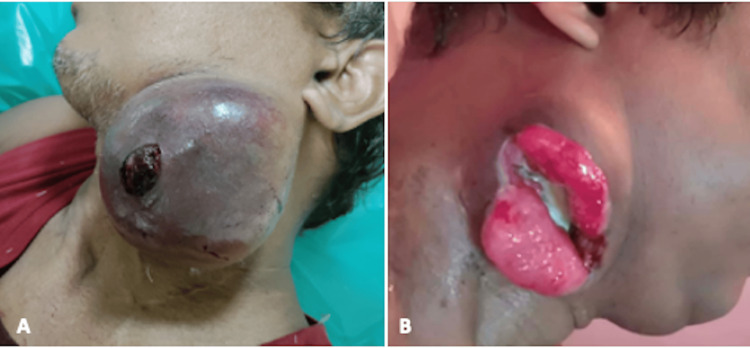
Clinical follow up images post embolisation status Photograph of the patient after one day (A) and two months (B) of embolization.

A few small arterial twigs were also seen arising directly from the ECA. Super-selective catheterisation of the occipital artery and superior thyroid artery was done using a microcatheter (Progreat, Terumo Intervention Systems, Tokyo, Japan), followed by distal embolisation with 300-500-micron polyvinyl alcohol (PVA) particles (Cook Medical, Bloomington, USA), which act at the microcirculation level. Finally, four metallic embolisation coils (Nester, Cook Medical, Bloomington, USA) were deployed along the entire course of ECA to block the small arterial twigs and avoid the development of collateral circulation in the future (Figure 2). Post-procedure contrast angiogram showed a completely knocked-off ECA with a reduced tumoral blush. Post-embolisation, the patient developed facial pain and required intravenous paracetamol. He was kept under observation. No bleeding episode was noticed, and he was discharged after seven days. After two months of embolisation, the size of the nodal mass was markedly reduced (Figure 3).

The patient remained alive at the eight-month follow-up, with no further bleeding episodes. The patient is now pain-free and on lenvatinib.

## Discussion

Patients with thyroid cancer may present with recurrent metastatic cervical lymph nodes, even after total thyroidectomy with lymph node dissection. Skin ulceration with bleeding from these lymph nodes is extremely rare. In our case, the patient presented with an ulcerated nodal neck mass with profuse bleeding. The bleeding was controlled by TAE as vascular involvement by the recurrent nodal mass created a nearly impossible situation for the surgeons, as access to the bleeding vessels was limited. TAE is routinely employed to manage life-threatening bleeding in head and neck trauma, intractable epistaxis, as well as bleeding resulting from locally advanced fungating or ulcerating head and neck malignancies, direct vascular invasion of the tumor, and carotid blowout syndrome, a rare yet devastating complication of radiation therapy in the neck [3].

Additionally, TAE is utilised as a preoperative embolisation technique for various vascular lesions of the head and neck, including arteriovenous malformations, glomus tumours, juvenile nasopharyngeal angiofibromas, and intracranial meningiomas, to reduce intraoperative blood loss. In TAE, bleeding is ceased by occluding the feeding artery with an embolisation agent [3,4]. Depending on the calibre of the vessel, the cause of bleeding, the type of vessel injury, and various other factors, a range of embolisation agents, including metallic coils, PVA particles, gel foam, and liquid embolic agents (such as glue or cyanoacrylate), may be utilised. Coils and gel foam are the most frequently used embolizing agents in blunt maxillofacial trauma or epistaxis. PVA particles are commonly used to reduce intraoperative blood loss for pre-operative embolisation of glomus tumours, juvenile nasopharyngeal angiofibromas, and intracranial meningiomas [5,6].

Glue, or cyanoacrylate, is frequently employed to embolize arteriovenous malformations. In bleeding caused by locally advanced ulcerating head and neck malignancies, PVA particles or coils are typically used as embolizing agents. Embolisation through the trans-arterial route is a minimally invasive and highly effective method to manage bleeding, especially in those nearly impossible conditions where surgical access to the bleeding vessels is limited because of vascular involvement [5,6]. Additionally, it allows selective closure of the culprit feeders with less chance of collateral development compared to surgical ligation. Moreover, via TAE, more long-lasting effects are achieved because of vascular occlusion at the microcirculation level [7-10].

Head and neck cancers can cause life-threatening bleeding due to advanced ulcers, invasion of nearby blood vessels, or carotid blowout syndrome. However, in our patient, the bleeding was from the ulcerated nodal neck mass. There were no features of carotid blowout syndrome or vascular invasion. Only one such case has been described in the literature regarding the palliative embolisation of the bleeding neck nodes by Naseer et al. in a patient with nasopharyngeal carcinoma [11]. Usually, minor and short-term complications are noted in these post-embolisation patients of head and neck cancers in the form of recurrent bleeding (5%), headache (18.4%), and facial oedema (7.8%) [3]. Other rare complications include ischemic stroke, loss of vision, or cranial nerve paresis [3,12]. Overall, TAE is a minimally invasive and safe procedure that has prolonged the lives of patients with recurrent tumours [13].

It should be noted that managing traumatic and non-traumatic causes of bleeding from the head and neck region is always a challenging scenario that requires a multidisciplinary approach involving interventional radiologists, otolaryngologists, oncologists, and neurosurgeons.

## Conclusions

Thyroid cancer patients may present with recurrent metastatic cervical lymph nodes, even after the standard treatment, including total thyroidectomy with lymph node dissection. Skin ulceration with bleeding from these recurrent metastatic lymph nodes is extremely rare. TAE is a minimally invasive and safe method with low morbidity and mortality rates, less chance of collateral development, and more long-lasting effects than surgical ligation, especially in those nearly impossible conditions where surgical access to the bleeding vessels is limited because of vascular involvement.
